# Lipid-Free Parenteral Nutrition Is Associated with an Increased Risk of Hepatic Dysfunction in Surgical Critically Ill Patients: A Retrospective Observational Study

**DOI:** 10.3390/healthcare9091096

**Published:** 2021-08-25

**Authors:** Shih-Chi Wu, Te-An Chen, I-Ju Tsai, Yu-Chun Wang, Han-Tsung Cheng, Chia-Wei Tzeng, Chia-Hao Hsu, Chih-Hsin Muo

**Affiliations:** 1 School of Medicine, China Medical University, Taichung 404, Taiwan; 2 Trauma and Emergency Center, China Medical University Hospital, Taichung 404, Taiwan; 3 Department of Surgery, China Medical University, Taichung 404, Taiwan; adblue818@gmail.com (T.-A.C.); traumawang@yahoo.com.tw (Y.-C.W.); howardcheng324@gmail.com (H.-T.C.); D11814@mail.cmuh.org.tw (C.-W.T.); D10759@mail.cmuh.org.tw (C.-H.H.); 4 Management Office for Health Data, China Medical University and Hospital, Taichung 404, Taiwan; hunch0815@hotmail.com (I.-J.T.); b8507006@gmail.com (C.-H.M.)

**Keywords:** hepatic function, intravenous fat emulsion, parenteral nutrition, trauma/acute care surgery, surgical critically ill

## Abstract

To evaluate the effects of lipid-free parenteral nutrition (PN) and various intravenous fat emulsions (IVFEs) on hepatic function in surgical critically ill trauma/acute care surgery patients. We retrospectively reviewed trauma/acute care surgery patients without admission hepatic disorder that received PN. The PN groups include lipid-free, soybean oil/medium-chain triglyceride, olive oil-based, and fish-oil contained PN. We excluded patients with (1) age <18 years, (2) without surgery, (3) preexisting liver injury/diseases, (4) hyperbilirubinemia at admission, (5) received more than one type of PN, and (6) repeated ICU episodes in the same hospitalization. Hepatic dysfunction was considered as serum total–bilirubin >6.0 mg/dL. The demographics, severity score, comorbidities, blood stream infection, and mortality were collected for analyses. The major outcome is hepatic function. We also performed analyses stratified by separated lipid doses (g/kg/day). A total of 249 patients were enrolled. There were no demographic differences among groups. The lipid-free PN group had a higher incidence of hepatic dysfunction and mortality. Compared to the lipid-free group, the other three IVFEs had significantly lower risks of hepatic dysfunction, while the olive oil-based group had a significantly lower risk of 30 and 90-day mortality. After being stratified by separating lipid doses, the soybean oils showed a decreasing trend of hepatic dysfunction and mortality with increased dosage. Fish oil >0.05 g/kg/day was associated with lower hepatic dysfunction incidences. Our findings suggest that, when compared to IVFEs, surgical critically ill patients with trauma/acute care surgery that received lipid-free PN are associated with an increased risk of hepatic dysfunction. In addition, the olive oil-based group had a significantly lower risk of mortality, while fish oil >0.05 g/kg/day was associated with lower incidences of hepatic dysfunction; however, further studies are warranted.

## 1. Introduction

Parenteral nutrition (PN) is essential in intestinal failure or intolerable to oral or enteral nutrition (EN) for prolonged periods [1,2]. IV fat emulsions (IVFEs) have been an important component of PN, which provide cellular energy and essential fatty acids [3,4]. The major components of fat emulsions may include soybean oil, olive oil, fish oil, and coconut oil-based IVFEs [4,5]. Moreover, PN and IVFEs are beneficial in undernourished patients or EN is not feasible [6]. However, which type of PN might be used in surgical critically ill patients was not clear [7,8,9].

Soybean-based IVLE are high in linoleic acid (about 50%), which is abundant in essential *n*-6 polyunsaturated fatty acids (PUFAs) and high phytosterol concentrations [3], which was thought to aggravate inflammatory immune response and have adverse outcomes [10]. Olive oil contains 85% nonessential *n*-9 oleic acid, about 4% essential *n*-6 linoleic acid, and is devoid of essential *n*-3 PUFAs. A common commercial olive oil-based IVLE is a mixture of 80% olive oil and 20% soybean oil [11]. Due to the large amount of *n*-9 monounsaturated fatty acids and dilution of the pro-inflammatory effect of *n*-6 PUFAs, olive oil-based IVLE is generally considered immunologically neutral [12].

Fish oil is more plentiful in *n*-3 FAs than plant-based oils, is rich in eicosapentanoic acid and docosahexaenoic acid, and is thought to have anti-inflammatory effects [13,14]. Compared to vegetal PN, fish oil-containing IVLE minimize liver function disturbance in hospitalized adult patients [15]. A common commercial fish oil-based IVLE product contains a mixture of soybean, MCT, olive, and fish oil [1]. Fish oil may be from different fish species, such as mackerel, herring, tuna, salmon, etc. [16].

PN-associated liver disease is a feared and life-threatening complication associated with parenteral nutrition dependence [17]. It is characterized by hepatobiliary disorders such as: cholestasis, steatosis, gallbladder sludge, and hepatic inflammation that might progress to cirrhosis and end-stage liver disease [18]. The incidence may vary from 40–60% in infants to 15–40% in adults [19]. The etiology may include liver-gut immunity, extensive intestinal disease, lack of enteral feeding, sepsis, and infections. [20], other etiologies may be associated with PN formulation or nutrient intake [21]. Studies showed that lipid restriction and replacing the soybean oil with parenteral fish-oil contained PN or emulsions of mixed-lipid sources are with lower incidences of PN complications in children and infants [22,23,24,25]. However, whether there were similar results in adult surgical critical ill patients is not clear.

Definitive diagnosis of PN-associated liver disease may include liver biopsy, which is not clinically practicable in all patients [4]. Instead, diagnosis is mainly based on bilirubin and liver enzyme levels, a serum direct bilirubin greater than 2.0 mg/dL is considered as an indicator [16,26]. Owing to the characteristics of this disease, it is central to evaluate the impact of PN and different IVLEs, as well as the source of lipid in PN admixtures, on hepatic function. Few studies have addressed the impact of different types of PNs on hepatic function in surgical critically ill patients with severe trauma/acute care surgery. Therefore, we aim to evaluate the effects of lipid-free PN and different IVFEs on hepatic function and the clinical outcomes in these patients.

## 2. Materials and Methods

From May 2013 to October 2017, we retrospectively reviewed the charts of patients with PN use in a surgical intensive care unit. We collected patients with torso trauma such as abdominal trauma with solid or hollow viscus injury, abdominal compartment syndrome, massive hemoperitoneum, pneumo/hemothorax, high grade liver or spleen injury, complicated pelvic fracture, and that patients underwent acute care surgery (such as hollow organ perforation with septic shock, bowel ischemic change, hepatobiliary obstruction) that were admitted for intensive care and received PN support during their ICU stay.

The exclusion criteria were: (1) received more than one type of IVFE, (2) without surgery, (3) with liver injury or preexisting liver diseases, biliary disorders or pancreatic cancer, (4) hyperbilirubinemia (serum total–bilirubin >2.0 mg/dL) at ICU admission, and (5) repeated ICU episodes during the same hospitalization.

Management of patients included team care, bundles of prevention/control of infection, and organ support protocols. Laboratory data were collected periodically. Sedation of patients was with non-propofol sedatives. As there were limited instructions for the selection of PN types in guidelines [7,8,9], there was no preference for the use of lipid-free PN (2-in-1 PN admixtures) [27] or IVFEs in the current series.

The supplementation of calories was 20–25 kcal/kg/day during the acute phase (48 h after ICU admission), and 30 kcal/kg/day during the post-acute phase (>4 days after ICU admission) [28]. If EN could not be established within 72–96 h after admission, the use of PN was considered [8,9]. However, in patients with preoperative malnutrition (e.g., BMI < 18.5 kg/m^2^) and those intolerable to enteral feeding, we started PN within 24 h of admission. The PN could be discontinued when EN reached a target of 60% nutritional requirements.

In this study, the IVFE products included soybean oil-based/medium/long-chain triglycerides (soybean oil/MCT) (Lipovenoes^®^ and Lipofundin^®^), olive oil-based (ClinOleic^®^) and mixed IVFE with fish oil (SMOF^®^). However, a 100% soybean oil-based IVFE was not available at our institution. Therefore, there were four groups of patients: lipid-free PN, soybean oil/MCT IVFE, olive oil-based IVFE, and fish oil-containing IVFE. The types and components of the IVFEs used are listed in Table 1.

For complying with the Personal Information Protection Act, the data abstracted from the chart contained no identification of patient information. All identifications of patients were replaced with surrogate numbers for research uses. All methods were performed in accordance with relevant guidelines and regulations. In addition, the need for written informed consent was waived by the Ethics Committee. This study was approved by the Research Ethics Committee at the China Medical University and Hospital (CMUH106-REC3-128).

## 3. Definition of Hepatic Dysfunction

There were lack of universal definitions of hepatic dysfunction/failure. Current diagnostic criteria of hepatic dysfunction are mostly based on laboratory serum bilirubin data, which may vary from more than 2.0 mg/dL (>34 μmol/L) to greater than 4 mg/dL (>70 μmol/L) [29,30,31]. We considered serum total bilirubin >6.0 mg/dL as an indicator of hepatic dysfunction, based on a sequential organ failure assessment (SOFA) score. The SOFA score defines serum total–bilirubin >6.0 mg/dL as grade III hepatic failure [32].

## 4. Assessment of Patient Severity

The evaluations of operative and perioperative status of severity are done with physiological severity scores, we used the Acute Physiology and Chronic Health Evaluation II (APACHE II) score, SOFA score, and the physiological and operative severity scores for the enumeration of mortality and morbidity scoring system (POSSUM) to evaluate the physiological status, surgical mortalities, and morbidities. The POSSUM score is often considered for surgical audits [33]. In addition, the Injury Severity Score (ISS) was used to assess the severity of trauma in patients.

## 5. Measurements

We performed analyses of demographics (including age, sex, and body weight), preexisting comorbidities (including sepsis, trauma, type II diabetes, hypertension, heart disease, chronic kidney disease, COPD, and received trans-arterial embolization), severity score of illness at admission (including admission APACHE II score, total POSSUM score, SOFA score, and ISS), days from PN start, days of PN use, days of EN establishment, initial laboratory data (including admission serum total-bilirubin, BUN, creatinine, serum albumin, serum lactate (mg/dL), and serum GPT), blood transfusion, hemodynamic instability on admission, received abdominal operation, renal replacement therapy, blood stream infection (BSI), ventilator days, length of stay, and time to hepatic dysfunction. Mortality was assessed at discharge, and 30 and 90 days. We also assessed the effects of IVFEs on the time to hepatic dysfunction and mortality, stratified by separated oil dosage (g/kg/day). In addition, we used median dosage for assessment because there was a lack of standard. 

## 6. Statistical Analysis

Due to data asymmetry, the differences in the demographic data and clinical characteristics were examined by Kruskal-Wallis tests for continuous variables and Chi-square tests/Fisher exact tests for categorical variables. Additionally, we used Cox regression models to analyze the risk of hepatic dysfunction or mortality among IVFE groups, and show the hazard ratios (HRs) with 95% confidence intervals (95% CIs). The association of different oil dosages with hepatic dysfunction and mortality among the different oil types was assessed. The separated oil dosages (g/kg/day) were grouped into two group, based on median dosage among oils.

The survival probability of mortality or cumulative incidence of hepatic dysfunction were plotted based on Cox’s model after being adjusted for significant variables (Table 1). All data management and analyses were performed using SAS 9.4 software package (SAS Institute, Cary, North Carolina. https://www.sas.com/zh_tw/home.html (accessed on 24 August 2021)) and the significance level was set at *p* < 0.05 under a two-tail test.

## 7. Result

**Figure 1:**
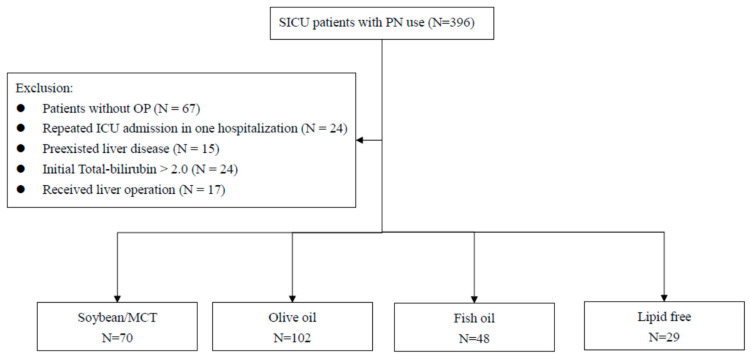
Flow chart for selecting study cohorts.

During this study period, 396 patients were collected. None of the patients had received more than one type of IVFE. We excluded 67 patients who did not have surgery, 17 patients with liver injury, 15 patients with pre-existing liver diseases, 24 patients with hyperbilirubinemia at ICU admission, and 24 patients with repeated ICU admissions during the same hospitalization. Therefore, a total of 249 patients were enrolled. There were 70 patients in the soybean oil-based/MCT group, 102 patients in olive oil-based IVFE group, 48 patients in fish oil-containing IVFE group, and 29 patients in the lipid-free group (Figure 1).

The mean age among the groups were between 60 to 70 years. The distributions of comorbidities, management and clinical parameters, laboratory data, and BSI among the groups were similar, except for the number of days of PN use (Table 2).

Table 3 showed that the lipid-free group had a higher mortality rate than other groups. In addition, when compared to lipid-free group, the olive oil-based IVFE group had a significantly lower risk of in-hospital mortality, and at 30 and 90 days (adjusted HR = 0.39, 0.25, and 0.41, respectively, with 95% CI = 0.18–0.84, 0.09–0.72, and 0.19–0.89).

The lipid-free group also showed the highest incidence of hepatic dysfunction (18.02 per 1000 person days), followed by the fish oil-containing IVFE, olive oil-based IVFE, and soybean/MCT group (8.25, 5.43, and 4.62 per 1000 person-days, Table 2). Moreover, these three IVFEs had significantly lower risks of hepatic dysfunction when compared to lipid-free group (adjusted HR = 0.25, 0.30, and 0.39, respectively, for soybean/MCT, olive oil-based IVFE, and fish oil-containing IVFE, Table 3). There was no significant hepatic dysfunction among the IVFE groups.

After being adjusted for days of PN use and stratified by separating the oil dosages (g/kg/day), there was a decreasing trend in the risk of time to hepatic dysfunction by increasing the oil dosage in soybean oil. However, olive oil ≤ median dosage (0.24 g/kg/day) had higher incidences of hepatic dysfunction than non-olive oil users, while olive oil > median dosage had a significantly lower risk of hepatic dysfunction than non-olive oil users (Table 4). The trends of fish oil were similar to those of olive oils at a median dosage about 0.05 g/kg/day, but without significance.

Table 5 shows the effects of different oil dosages on time to in-hospital mortality, stratified by separated oil dosages. Unexpectedly, soybean oil had a significantly lower risk when compared to non-soybean oil users (HR = 0.51 in ≤ median and 0.44 in > median, 95% CI = 0.25–1.04 and 0.21–0.91 for g/kg/day). Moreover, patients who received fish oil dosages ≤ median (0.05 g/kg/day) had a higher risk of mortality, while >0.05 g/kg/day had a lower risk of mortality; however, this was without significance.

**Figure 2:**
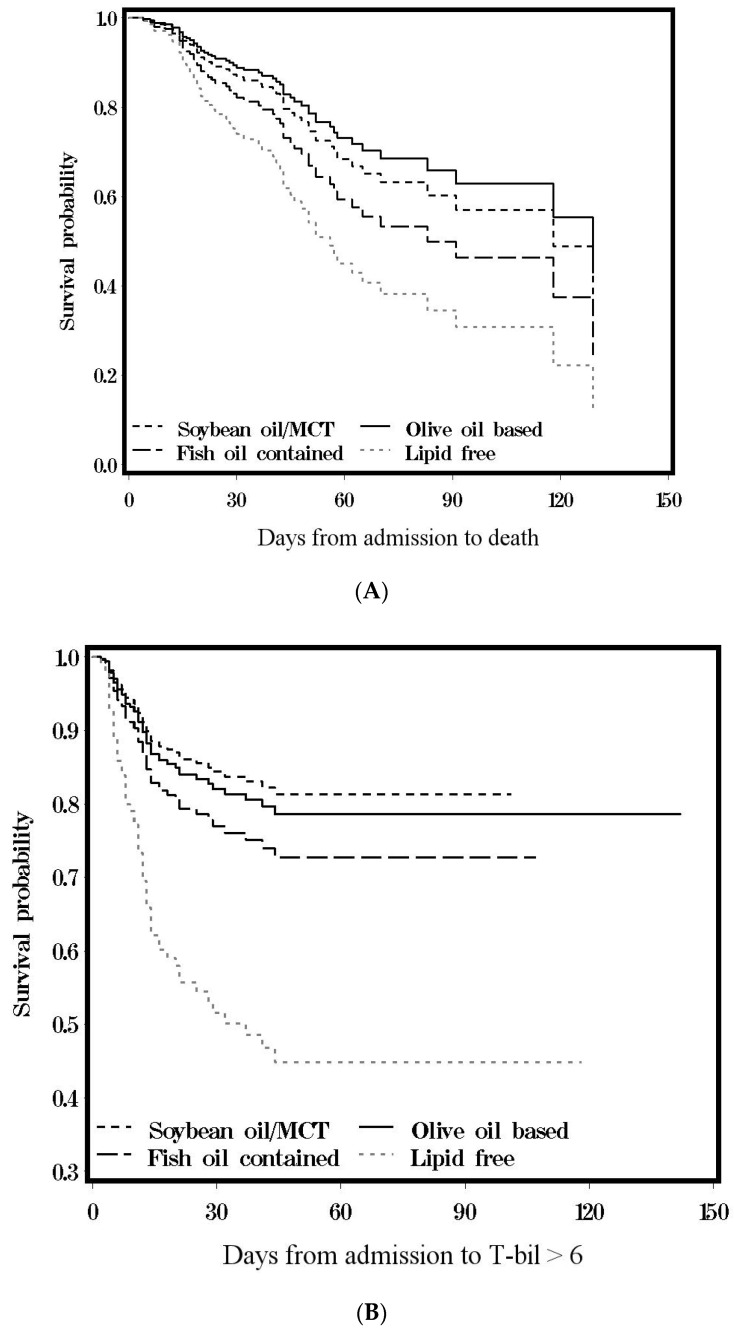
(**A**) Cumulative incidence of mortality in Cox’s model; (**B**) cumulative incidence of T-bilirubin > 6.0 mg/dL in Cox’s model.

Figure 2A shows the curve of cumulative mortalities among the four groups. The lipid-free group was the highest (98.4%), followed by fish oil-containing (75.4%), soybean/MCT group (64.1%), and olive oil-based (57.1%).

Figure 2B shows the cumulative incidence of hepatic dysfunction among groups. The lipid-free group was the highest (55.2%), followed by fish oil-containing (27.3%), olive oil-based (21.4%), and soybean/MCT group (18.7%).

## 8. Discussion

The characteristics of different subgroups of critically ill patients may be similar but are not universally identical [34]. For example, expert opinions support the use of olive oil and fish oil in nutrition support in surgical and non-surgical ICU patients, but considers further research as required to provide more robust evidence [35]. Others suggest holding or limiting soy-based IVLE during the first week of PN therapy in critically ill patients [36,37]; however, these suggestions may be limited to a selected group and may not be generalizable to a broader group of patients [5]. In addition, surgical critically ill patients with severe trauma/acute care surgery were more susceptible to acute stress, shock, coagulopathy, and multi-organ failure. [38,39]. Therefore, it is likely that recognized therapeutic concepts and modalities might yield different outcome in these patients.

Because there were confounding factors for the development of hepatic dysfunction in critical illness, such as previous comorbidities, preexisting cirrhosis/hepatic injuries, injury severity, shock liver, sepsis and infection, blood transfusion, and multiple organ failure [40], therefore, we excluded patients with liver injury, preexisting liver diseases, and hyperbilirubinemia at ICU admission. In the current series, we use serum total bilirubin level > 6.0 mg/dL as an indicator for hepatic dysfunction because more than 2.0 mg/dL could be easily reached in critically ill patients.

Regardless of the shortest use of interval, the lipid-free PN group had a higher incidence of hepatic dysfunction and mortality among the groups. After being adjusted for days of PN use, the olive oil-based group had a significantly lower risk of 30- and 90-day mortality in comparing with the lipid-free group, while there was no significance for soybean/MCT and fish oil-containing IVFEs. Moreover, there was no significant risk of hepatic dysfunction and mortality among the three IVFE groups (Table 3). This result might highlight the importance of essential fatty acids in surgical critical patients.

Although lipids may not play a critical role in the mortality of surgical critical patients, a likely explanation of the lowest risk of mortality for olive oil-based IVFE might be attributed to its immune feature. As is known, there were immune disturbances in severe trauma/acute care surgery patients [41]. Studies showed that high olive oil IVFE concentrations may have less impact on host immune response than soybean oil-based or soybean/MCT IVFEs [2,42]. In the current series, olive oil dosages more than 0.24 g/kg/day had a significantly lower risk of hepatic dysfunction than non-olive oil use, which indicated that the immune-neutral effect of olive oil-based IVFE might play an important role.

The fish oil was had anti-inflammatory and anticoagulant effects. There was a beneficial effect of fish oil-containing IVFE in critical illness and hepatic dysfunction treatment, especially in pediatric patients [22,43]. Furthermore, the dosage of fish oil could be essential, the study showed a reduced length of stay and ICU stay with fish oil >0.05 g/kg/day, while fish oil >0.1 and <0.2 g/kg/day was associated with reduced mortality in critically ill patients receiving total parenteral nutrition [44]. In the current study, though without significance, we found that fish oil dosages <0.05 g/kg/day were with a higher risk of hepatic dysfunction and mortality, while a dosage >0.05 g/kg/day had a lower risk when compared to non-fish oil use (Table 4 and Table 5), indicating the importance of fish oil dosage.

The soybean oil IVLE was noted as having higher contents of phytosterols and polyunsaturated fatty acids, which may be associated with negative impacts on the immunological status in the critically ill [45]. However, when stratified by separate lipid doses (g/kg/day), soybean oils showed a decreasing trend of hepatic dysfunction and mortality with increased oil dosages (Table 4 and Table 5). Though there were a limited number of patients, this result might reflect, at least in part, an undiscovered role of soybean lipid supplementation in this patient group. We assume that there may be an increased requirement for *n*-6 essential fatty acids in such patients. Further studies, however, are warranted.

In summary, our study showed that, in surgical critically ill patients with trauma/acute care surgery, the use of lipid-free PN is associated with an increased incidence of hepatic dysfunction. Olive oil-based IVFE showed a lower risk of hepatic dysfunction and mortality, which might be attributed to its immune-neutral effect. When stratified by separated dosages, soybean oils, unexpectedly, showed the lowest incidence of hepatic dysfunction. Moreover, the dosage of fish oil appears crucial. However, further studies are warranted.

## 9. Limitation of the Study

The strengths of this study include a specific study population, reliable diagnoses and a high follow-up rate. However, we recognize that certain limitations existed. First, the sample size was not large. Second, due to its retrospective nature, lack of randomization may give rise to probable bias in case selection, which might restrict our analytical conclusions. Third, because there were multi-factorial characteristics in surgical critically ill patients with severe trauma/acute care surgery, it is difficult to collect all of the related data in this study. Therefore, evaluation of the physiological status and severity of these patients was done using physiological scores rather than detailed clinical parameters. Fourth, the criteria for the use and type of PN were not fully quantified, which might have subjective bias. Therefore, further multi-center randomized studies for this specific patient group are warranted with predefined enrollment criteria for a better understanding of this issue.

## 10. Conclusions

Our findings suggest that, when compared with IVFEs, surgical critically ill patients with trauma/acute care surgery who received lipid-free parenteral nutrition are associated with an increased risk of hepatic dysfunction. In addition, the olive oil-based group had a significantly lower risk of mortality, while fish oil >0.05 g/kg/day was associated with lower incidences of hepatic dysfunction. However, based on its retrospective nature and limited sample size, further studies are warranted.

## Figures and Tables

**Table 1:** Intravenous lipid emulsions used in current study population.

Characteristics	Soybean Oil/MCT		Olive Oil Based	Fish Oil Contained
Commercial Products	Lipovenoes^®^	Lipofundin^®^	ClinOleic 20%^®^	SMOF^®^
Lipid source (%)				
Soybean oil	50	50	20	30
MCT	50	50	0	30
olive oil	0	0	80	25
Fish oil	0	0	0	15

MCT: medium-chain triglyceride, LCT: long-chain triglyceride.

**Table 2:** Demographics and clinical characteristics.

Variable	Soybean Oil /MCT (*N* = 70)	Olive Oil Based (*N* = 102)	Fish Oil Contained (*N* = 48)	Lipid Free (*N* = 29)	*p*-Value
Demographic					
Age (y), mean (SD)	66.2 (18.8)	69.0 (15.8)	66.5 (15.9)	63.1 (18.1)	0.4239
Sex, *n* (%)					
Female	32 (45.71)	47 (46.08)	14 (29.17)	12 (41.4)	0.2275
Male	38 (54.29)	55 (53.92)	34 (70.83)	17 (58.6)	
Body weight (kg), mean (SD)	60.1 (15.3)	57.9 (12.6)	62.8 (12.5)	62.2 (18.2)	0.1375
Clinical, mean (SD)					
Severity score of illness					
Admission APACHEII score	16.5 (7.27)	17.9 (8.26)	17.0 (7.73)	18.82 (8.47)	0.5243
SOFA score	3.88 (2.54)	3.91 (2.97)	3.52 (2.67)	5.45 (3.37)	0.0676
Total POSSUM score	46.7 (9.92)	44.9 (9.78)	45.0 (10.7)	50.62 (11.80)	0.0728
ISS ^†^	28.3 (12.7)	21.8 (12.5)	22.3 (12.1)	30.8 (3.76)	0.2171
Days of PN start, mean (SD)	3.74 (5.53)	3.59 (3.78)	3.46 (5.24)	4.21 (4.45)	0.4031
Days of EN establish, mean (SD)	3.58 (2.13)	4.18 (3.45)	5.12 (3.95)	4.39 (4.44)	0.1279
Days of PN use, mean (SD)	12.1 (10.7)	14.1 (17.7)	18.3 (18.1)	8.21 (6.50)	0.0008
Admission biochemical variables, mean (SD)					
Admission initial serum T-bilirubin	0.92(0.50)	0.91(0.47)	1.07(0.51)	1.14 (0.60)	0.1286
BUN	38.1 (30.2)	34.9 (26.5)	32.7 (30.5)	30.9 (27.3)	0.2480
Creatinine	2.20 (2.05)	2.21 (2.28)	1.87 (1.86)	2.45 (2.77)	0.5874
Serum albumin	2.88 (0.66)	2.9 (0.65)	2.66 (0.68)	2.74 (0.64)	0.3543
Serum lactate (mg/dL)	42.8 (34.7)	38.6 (29.7)	39.5 (36.9)	49.9 (47.2)	0.6090
Serum GPT	67.6 (175)	51.8 (104)	33.1 (26.6)	97.6 (136)	0.1257
Blood transfusion	8.14 (11.3)	5.85 (7.76)	8.65 (17.9)	12.5 (16.5)	0.3670
Comorbidity, *n* (%)					
Sepsis	15 (21.43)	20 (19.61)	13 (27.08)	4 (13.8)	0.5802
Trauma	13 (18.57)	17 (16.67)	7 (14.58)	6 (20.7)	0.8668
Type II diabetes	36 (51.43)	51 (50.00)	28 (58.33)	16 (55.2)	0.7952
Hypertension	30 (42.86)	46 (45.10)	17 (35.42)	12 (41.4)	0.7345
Heart disease	7 (10.00)	12 (11.76)	6 (12.50)	3 (10.3)	0.9815
Chronic kidney disease	14 (20.00)	21 (20.59)	6 (12.50)	6 (20.7)	0.6617
COPD	1 (1.43)	3 (2.94)	1 (2.08)	2 (6.90)	0.5061
Malignancy	12 (17.14)	21 (20.59)	11 (22.92)	3 (10.3)	0.5449
Post TAE	6 (8.57)	7 (6.86)	2 (4.17)	4 (13.8)	0.4554
Hemodynamic unstable on admission, *n* (%)	26 (37.14)	36 (35.29)	21 (43.75)	13 (44.8)	0.6719
Received abdominal operation, *n* (%)	64 (91.43)	91 (89.22)	45 (93.75)	25 (86.2)	0.6872
Vasopressor use at ER, *n* (%)	12 (17.14)	15 (14.71)	7 (14.58)	9 (31.0)	0.2071
Received CVVH, *n* (%)	5 (7.14)	15 (14.71)	7 (14.58)	7 (24.1)	0.1478
Received HD, *n* (%)	6 (8.57)	15 (14.71)	5 (10.42)	8 (27.6)	0.0775
Blood stream infection, *n* (%)	32 (45.71)	34 (33.33)	18 (37.50)	12 (41.4)	0.4221
Length of stay (day), mean (SD)	35.6 (26.1)	37.6 (28.5)	40.0 (28.0)	37.0 (29.4)	0.7693
Duration of ventilator days, mean (SD)	14.6 (14.4)	19.6 (21.6)	17.6 (15.3)	21.8 (23.6)	0.2495

*p*-values were calculated by Kruskal-Wallis tests for continuous variables and Chi-square tests/Fisher exact tests for categorical variables. MCT: medium-chain triglyceride, TAE: trans-arterial embolization, CVVH: continuous venovenous hemofiltration, HD: hemodialysis; ^†^ ISS score only for trauma patients.

**Table 3:** Effect of intravenous fat emulsions on time to mortality or T-bilirubin > 6.0 mg/dL.

Outcome	No. of Event	Person-Days	Incidence ^†^	HR (95% CI)	*p*-Value	HR (95% CI)	*p*-Value
Mortality at discharge Soybean oil/MCT	13	2494	5.21	0.48 (0.22–1.05)	0.0665	Ref.	
Olive oil-based	17	3839	4.43	0.39 (0.18–0.84)	0.0156	0.82 (0.39–1.69)	0.5828
Fish oil containing	13	1918	6.78	0.65 (0.29–1.47)	0.3043	1.32 (0.60–2.91)	0.4854
Lipid free	12	1074	11.17	Ref.			
30-day mortality							
Soybean oil/MCT	9	1623	5.55	0.53 (0.20–1.38)	0.1899	Ref.	
Olive oil-based	6	2371	2.53	0.25 (0.09–0.72)	0.0102	0.47 (0.17–1.31)	0.1488
Fish oil containing	6	1204	4.98	0.55 (0.18–1.65)	0.2888	1.03 (0.36–2.93)	0.9587
Lipid free	8	647	12.36	Ref.			
90-day mortality							
Soybean oil/MCT	13	2433	5.34	0.53 (0.24–1.19)	0.1223	Ref.	
Olive oil-based	15	3691	4.06	0.41 (0.19–0.89)	0.0242	0.76 (0.36–1.60)	0.4671
Fish oil containing	13	1823	7.13	0.76 (0.33–1.75)	0.5222	1.42 (0.65–3.10)	0.3840
Lipid free	11	1031	10.67	Ref.			
T-bilirubin > 6 mg/dL							
Soybean oil/MCT	10	2163	4.62	0.25 (0.11–0.59)	0.0014	Ref.	
Olive oil-based	18	3312	5.43	0.30 (0.14–0.63)	0.0015	1.15 (0.52–2.51)	0.7335
Fish oil containing	12	1455	8.25	0.39 (0.17–0.89)	0.0248	1.53 (0.66–3.57)	0.3230
Lipid free	12	666	18.02	Ref.			

^†^ per 1000 person days; MCT: medium-chain triglyceride; adjusted for days of PN use.

**Table 4:** Effect of different lipids on time to T-bilirubin > 6.0 mg/dL stratified by separated oil dosages (g/kg/day).

Variable	*N*	No. of Event	Person-Days	Incidence ^†^	HR (95% CI) *	*p*-Value
Soybean						
None	29	12	666	18.02	Ref.	
≤median (0.15 g/kg/day)	110	31	3159	9.81	0.52 (0.26–1.02)	0.0583
>median	110	9	3771	2.39	0.14 (0.06–0.32)	<0.0001
MCT						
None	99	23	2449	9.39	Ref.	
≤median (0.22 g/kg/day)	75	23	2732	8.42	0.96 (0.50–1.85)	0.9107
>median	75	6	2415	2.48	0.28 (0.11–1.69)	0.0057
Olive oil						
None	88	20	2379	8.41	Ref.	
≤median (0.24 g/kg/day)	80	25	2704	9.25	1.10 (0.59–2.04)	0.7709
>median	81	7	2513	2.79	0.34 (0.14–0.80)	0.0133
Fish oil						
None	172	33	4722	6.99	Ref.	
≤median (0.05 g/kg/day)	38	16	1531	10.45	1.72 (0.88–3.35)	0.1139
>median	39	3	1343	2.23	0.33 (0.10–1.06)	0.0628

^†^ per 1000 person days; MCT: medium-chain triglyceride; * adjusted for days of PN use.

**Table 5:** Effect of different lipids on time to in-hospital mortality stratified by separated oil dosages (g/kg/day).

Variable	*N*	No. of Event	Person-Days	Incidence ^†^	HR (95% CI) *	*p*-Value
Soybean						
None	29	12	1074	11.17	Ref.	
≤median (0.15 g/kg/day)	110	24	4299	5.58	0.51 (0.25–1.04)	0.0641
>median	110	19	3952	4.81	0.44 (0.21–0.91)	0.0289
MCT						
None	99	21	3183	6.60	Ref.	
≤median (0.22 g/kg/day)	75	20	3664	5.46	0.82 (0.40–1.66)	0.5748
>median	75	14	2478	5.65	0.87 (0.44–1.73)	0.6983
Olive oil						
None	88	23	3046	7.55	Ref.	
≤median (0.24 g/kg/day)	80	17	3629	4.68	0.60 (0.31–1.16)	0.1286
>median	81	15	2650	5.66	0.76 (0.40–1.47)	0.4161
Fish oil						
None	172	33	5799	5.69	Ref.	
≤median (0.05 g/kg/day)	38	15	2078	7.22	1.39 (0.70–2.73)	0.3464
>median	39	7	1448	4.83	0.91 (0.40–2.07)	0.8212

^†^ per 1000 person days; MCT: medium-chain triglyceride; * adjusted for days of PN use.

## Data Availability

Please contact author for data requests.

## References

[1] Raman M., Almutairdi A., Mulesa L., Alberda C., Beattie C., Gramlich L. (2017). Parenteral Nutrition and Lipids. Nutrients.

[2] Bielawska B., Allard J.P. (2017). Parenteral Nutrition and Intestinal Failure. Nutrients.

[3] Calder P.C., Jensen G.L., Koletzko B.V. (2010). Lipid emulsions in parenteral nutrition of intensive care patients: Current thinking and future directions. Intensive Care Med..

[4] Fell G.L., Nandivada P., Gura K.M., Puder M. (2015). Intravenous Lipid Emulsions in Parenteral Nutrition. Adv. Nutr..

[5] Edmunds C.E., Brody R.A., Parrott J.S. (2014). The effects of different IV fat emulsions on clinical outcomes in criti-cally ill pa-tients. Crit. Care Med..

[6] Braga M., Ljungqvist O., Soeters P., Fearon K., Weimann A., Bozzetti F. (2009). ESPEN Guidelines on Parenteral Nutrition: Surgery. Clin. Nutr..

[7] McClave S.A., Taylor B.E., Martindale R.G., Warren M.M., Johnson D.R., Braunschweig C., McCarthy M.S., Davanos E., Rice T.W., Cresci G.A. (2016). Guidelines for the Provision and Assessment of Nutrition Support Therapy in the Adult Critically Ill Patient: Society of Critical Care Medicine (SCCM) and American Society for Parenteral and Enteral Nutrition (A.S.P.E.N.). J. Parenter Enter. Nutr..

[8] Weimann A., Braga M., Carli F., Higashiguchi T., Hübner M., Klek S., Laviano A., Ljungqvist O., Lobo D.N., Martindale R. (2017). ESPEN guideline: Clinical nutrition in surgery. Clin. Nutr..

[9] Singer P., Blaser A.R., Berger M.M., Alhazzani W., Calder P.C., Casaer M.P., Hiesmayr M., Mayer K., Montejo J.C., Pichard C. (2019). ESPEN guideline on clinical nutrition in the intensive care unit. Clin. Nutr..

[10] Miloudi K., Comte B., Rouleau T., Montoudis A., Levy E., Lavoie J.-C. (2012). The mode of administration of total parenteral nutrition and nature of lipid content influence the generation of peroxides and aldehydes. Clin. Nutr..

[11] Vanek V.W., Seidner D.L., Allen P. (2012). Novel Nutrient Task Force, Intravenous Fat Emulsions Workgroup; Amer-ican Socie-ty for Parenteral and Enteral Nutrition (A.S.P.E.N.) Board of Directors: A.S.P.E.N. position paper: Clinical role for alternative intravenous fat emulsions. Nutr. Clin. Pract..

[12] Waitzberg D.L., Torrinhas R.S., Jacintho T.M. (2006). New Parenteral Lipid Emulsions for Clinical Use. J. Parenter Enter. Nutr..

[13] De Nardi L., Bellinati-Pires R., Torrinhas R.S., Bacchi C.E., Arias V., Waitzberg D.L. (2008). Effect of fish oil containing parenteral lipid emulsions on neu-trophil chemotaxis and resident-macrophages’ phagocytosis in rats. Clin. Nutr..

[14] Calder P.C. (2010). Omega-3 Fatty Acids and Inflammatory Processes. Nutrients.

[15] Llop-Talaveron J.M., Badia-Tahull M.B., Leiva-Badosa E., Ramon-Torrel J.M. (2017). Parenteral fish oil and liver function tests in hospi-talized adult patients receiving parenteral nutrition: A propensity score-matched analysis. Clin. Nutr..

[16] Kelly D.A. (2006). Intestinal Failure–Associated Liver Disease: What Do We Know Today?. Gastroenterology.

[17] Wales P.W., Allen N., Worthington P., George D., Compher C., American Society for Parenteral and Enteral Nutrition (2014). Clinical guidelines: Support of pediatric patients with intestinal failure at risk of parenteral nutrition-associated liver disease. J. Parenter Enter. Nutr..

[18] Nandivada P., Carlson S.J., Chang M.I., Cowan E., Gura K.M., Puder M. (2013). Treatment of Parenteral Nutrition-Associated Liver Disease: The Role of Lipid Emulsions. Adv. Nutr..

[19] Tillman E.M. (2012). Review and Clinical Update on Parenteral Nutrition–Associated Liver Disease. Nutr. Clin. Pr..

[20] Xu Z.-W., Li Y.-S. (2012). Pathogenesis and treatment of parenteral nutrition-associated liver disease. Hepatobiliary Pancreat. Dis. Int..

[21] Kumpf V.J. (2006). Parenteral Nutrition-Associated Liver Disease in Adult and Pediatric Patients. Nutr. Clin. Pr..

[22] Rollins M.D., Ward R.M., Jackson W.D., Mulroy C.W., Spencer C.P., Ying J., Greene T., Book L.S. (2013). Effect of decreased parenteral soybean lipid emulsion on hepatic function in infants at risk for parenteral nutrition-associated liver disease: A pilot study. J. Pediatr. Surg..

[23] Nandivada P., Fell G.L., Gura K.M., Puder M. (2016). Lipid emulsions in the treatment and prevention of parenteral nutrition-associated liver disease in infants and children. Am. J. Clin. Nutr..

[24] Sanchez S., Braun L.P., Mercer L.D., Sherrill M., Stevens J., Javid P.J. (2013). The effect of lipid restriction on the prevention of parenteral nutrition-associated cholestasis in surgical infants. J. Pediatr. Surg..

[25] Badia-Tahull M.B., Talaveron J.L., Leiva-Badosa E. (2015). Impact of intravenous lipid emulsions on liver function tests: Contribution of parenteral fish oil. Nutrition.

[26] Buchman A.L., Iyer K., Fryer J. (2005). Parenteral nutrition-associated liver disease and the role for isolated intestine and intestine/liver transplantation. Hepatology.

[27] Slattery E., Rumore M.M., Douglas J.S., Seres D.S. (2014). 3-in-1 vs 2-in-1 parenteral nutrition in adults: A review. Nutr. Clin. Pract..

[28] Patkova A., Josková V., Havel E., Kovařík M., Kuchařová M., Zadak Z., Hronek M. (2017). Energy, Protein, Carbohydrate, and Lipid Intakes and Their Effects on Morbidity and Mortality in Critically Ill Adult Patients: A Systematic Review. Adv. Nutr..

[29] Pastor C.M., Suter P.M. (1999). Hepatic hemodynamics and cell functions in human and experimental sepsis. Anesth. Analg..

[30] Sands K.E., Bates D.W., Lanken P.N., Graman P.S., Hibberd P.L., Kahn K.L., Parsonnet J., Panzer R., Orav E.J., Snydman D. (1997). Epidemiology of Sepsis Syndrome in 8 Academic Medical Centers. JAMA.

[31] Levy M.M., Fink M.P., Marshall J.C. (2003). 2001 SCCM/ESICM/ACCP/ATS/SIS International Sepsis Definitions Con-ference. Crit. Care Med..

[32] Vincent J.L., Moreno R., Takala J. (1996). The SOFA (Sepsis-related Organ Failure Assessment) score to describe or-gan dysfunction/failure. On behalf of the Working Group on Sepsis-Related Problems of the European Society of Intensive Care Medicine. Intensive Care Med..

[33] Mohil R.S., Bhatnagar D., Bahadur L. (2004). POSSUM and P-POSSUM for risk-adjusted audit of patients undergo-ing emergency laparotomy. Br. J. Surg..

[34] Parizkova R., Cerny V., Dostal P. (2001). The cost in different subgroups of critically ill patients: A multicentric study in Czech Republic. Crit. Care.

[35] Calder P.C., Adolph M., Deutz N.E. (2018). Lipids in the intensive care unit: Recommendations from the ESPEN Ex-pert Group. Clin Nutr..

[36] Taylor B.E., McClave S.A., Martindale R.G., Warren M.M., Johnson D.R., Braunschweig C., McCarthy M.S., Davanos E., Rice T.W., Cresci G.A. (2016). Guidelines for the provision and assessment of nutrition support therapy in the adult critically ill patient: Society of Critical Care Medicine (SCCM) and American Society for Parenteral and Enteral Nutrition (A.S.P.E.N.). Crit. Care Med..

[37] Critical Care Nutrition Canadian Clinical Practice Guidelines, Composition of Parenteral Nutrition: Type of Lipids 2013. www.criticalcarenutrition.com.

[38] Weimann A., Singer P. (2013). Avoiding underfeeding in severely ill patients. Lancet.

[39] Vogel J.A., Liao M.M., Hopkins E. (2014). Prediction of postinjury multiple-organ failure in the emergency depart-ment: Development of the Denver Emergency Department Trauma Organ Failure score. J. Trauma Acute Care Surg..

[40] Soultati A., Dourakis S.P. (2005). Liver dysfunction in the intensive care unit. Ann. Gastroenterol..

[41] Gentile L.F., Cuenca A.G., Efron P.A. (2012). Persistent inflammation and immunosuppression: A common syndrome and new horizon for surgical intensive care. J. Trauma Acute Care Surg..

[42] Buenestado A., Cortijo J., Sanz M.J. (2006). Olive oil-based lipid emulsion’s neutral effects on neutrophil functions and leukocyte-endothelial cell interactions. JPEN J. Parenter Enter. Nutr..

[43] Puder M., Valim C., Meisel J.A., Le H.D., de Meijer V.E., Robinson E.M., Zhou J., Duggan C., Gura K.M. (2009). Parenteral Fish Oil Improves Outcomes in Patients With Parenteral Nutrition-Associated Liver Injury. Ann. Surg..

[44] Heller A.R., Rößler S., Litz R.J., Stehr S.N., Heller S.C., Koch R., Koch T. (2006). Omega-3 fatty acids improve the diagnosis-related clinical outcome. Crit. Care Med..

[45] Manzanares W., Langlois P.L., Hardy G. (2016). Intravenous lipid emulsions in the critically ill: An update. Curr. Opin. Crit. Care.

